# Revisiting bone healing strategies: the potential of reverse dynamization in veterinary orthopedics

**DOI:** 10.3389/fvets.2026.1811517

**Published:** 2026-07-20

**Authors:** Anas Datoussaid, Marc Balligand, Pierre P. Picavet

**Affiliations:** 1 Department of Civil and Environmental Engineering, Massachusetts Institute of Technology, Cambridge, MA, United States; 2 Department of Companion Animal Clinical Sciences, Faculty of Veterinary Medicine, University of Liège, Liège, Belgium; 3 Department of Clinical Sciences, College of Veterinary Medicine, Kansas State University, Manhattan, KS, United States

**Keywords:** bone regeneration, companion animals, fracture healing, reverse dynamization, veterinary orthopedics

## Abstract

Reverse dynamization is an emerging orthopedic strategy that modulates fixation stiffness throughout fracture healing to better align the mechanical environment with the evolving biological requirements of tissue repair. In contrast to traditional dynamization, which typically begins with rigid fixation followed by progressive destabilization, reverse dynamization permits controlled early interfragmentary motion before increasing construct stiffness during later stages of healing. This review examines the biological and biomechanical foundations of reverse dynamization and evaluates its potential relevance for companion animal orthopedic surgery. Current preclinical evidence from rodent, ovine, and caprine models suggests that reverse dynamization can accelerate callus formation, improve tissue organization, enhance vascularization, and increase the mechanical competence of healing bone compared with static or conventionally dynamized fixation strategies. These findings support the concept that staged modulation of fixation stiffness may better reflect the changing mechanobiological requirements of fracture repair. Particular attention is given to veterinary-specific considerations, including quadrupedal locomotion, variable postoperative loading, reliance on secondary bone healing, and the potential applicability of adaptive fixation systems such as external fixators and dynamic implant constructs. However, the available evidence remains largely restricted to controlled experimental models and is limited by small sample sizes, sparse independent replication, and the absence of clinical studies in veterinary patients. Reverse dynamization should therefore be regarded as a promising mechanobiological concept rather than an established clinical strategy in veterinary orthopedics. Further veterinary-specific investigations are required to define optimal mechanical parameters, evaluate clinical feasibility, and determine whether the benefits observed in experimental models translate to routine fracture management in companion animals.

## 1 Introduction

Bone healing is a physiological process through which injured bone restores its biological integrity and mechanical function through a tightly coordinated interaction between cellular activity and biomechanical regulation (1, 2). Bone tissue continuously adapts to its mechanical environment, and the local loading conditions at a fracture site play a decisive role in determining both the pathway and efficiency of healing (3, 4). Consequently, fracture repair does not follow a single universal pathway but is largely influenced by construct stability and interfragmentary motion.

Two principal modes of bone healing are classically distinguished: primary (direct) and secondary (indirect) bone healing. Primary bone healing requires near-absolute interfragmentary stability and precise anatomical reduction, allowing direct cortical remodeling without callus formation (5, 6). In routine veterinary orthopedic practice, such conditions are rarely achieved or intentionally pursued, and the majority of fractures in companion animals therefore heal through secondary bone healing. This indirect pathway is characterized by callus formation and progresses through fibrous and cartilaginous stages before endochondral ossification restores cortical continuity (2, 6). Within this context, controlled interfragmentary motion represents an important regulator of tissue differentiation and callus maturation (3, 7).

Despite this mechanobiological understanding, fracture fixation strategies have traditionally emphasized initial construct rigidity to maintain alignment and stability. While rigid osteosynthesis has improved outcomes in many situations, excessive stiffness may limit early mechanical stimulation and has been associated with delayed union, nonunion, or implant-related complications (3, 8). To mitigate these limitations, dynamization was introduced as a strategy based on secondary de-rigidification of the fixation construct, involving a controlled reduction in construct stiffness during the course of healing to increase interfragmentary motion. Although delayed dynamization has been shown to influence callus development in experimental and clinical settings, its application remains variable. In particular, challenges related to timing, magnitude, and directional control of construct de-rigidification have limited its predictable use in veterinary practice (9–11).

Reverse dynamization represents a conceptual shift from this approach. Rather than beginning with maximal rigidity, it proposes an initially more permissive mechanical environment during early healing, followed by a transition to increased stiffness as mineralization and structural consolidation progress (12–14). While this strategy has shown promising results in experimental and preclinical animal models (12, 15–17), its relevance and applicability in veterinary orthopedic surgery remain largely unexplored.

While reverse dynamization has been reviewed previously in the context of human orthopedics and preclinical animal models, no synthesis to date has specifically examined its potential relevance, challenges, and translational prospects for companion animal orthopedic surgery. The distinct biomechanics of quadrupedal gait, the reliance on secondary bone healing in most veterinary fractures, the unpredictability of postoperative loading associated with variable owner compliance with postoperative restrictions, and the wide range of patient sizes collectively create a unique mechanobiological and clinical context that warrants independent evaluation. The aim of this review is to synthesize the current biological and biomechanical evidence related to reverse dynamization and to critically evaluate its potential relevance, limitations, translational challenges, and knowledge gaps within veterinary orthopedics.

## 2 Biological and mechanical foundations

Bone healing is a highly orchestrated process governed by tightly coupled biological and mechanical factors. Following fracture, repair progresses through overlapping phases that include inflammation, callus formation, and subsequent maturation and remodeling, during which newly formed tissue gradually restores mechanical competence (2). Throughout this process, the local mechanical environment plays a decisive role in determining both the rate of healing and the type of tissue formed at the fracture site.

### 2.1 Mechanical regulation of tissue differentiation

Fracture healing does not occur in a mechanically neutral environment. Interfragmentary motion and strain strongly influence cellular behavior and tissue differentiation throughout the repair process. Classical mechanobiological concepts describe how different levels of strain promote distinct tissue phenotypes: excessive motion favors fibrous tissue formation, moderate strain supports chondrogenesis, and low strain facilitates osteogenesis (18).

At the cellular level, mechanotransduction plays a central role in this process. Mechanical stimuli influence mesenchymal stem cell fate by regulating proliferation, differentiation, and extracellular matrix production (19, 20). Osteocytes act as key mechanosensors, translating mechanical loading into biochemical signals that regulate osteoblast and osteoclast activity, thereby coordinating bone formation and resorption during healing (21, 22).

These principles are consistent with Wolff’s law and later strain-based theories such as Frost’s mechanostat, which propose that bone adapts its structure according to the mechanical environment it experiences. Frost proposed that bone adaptation and remodeling are regulated by discrete strain thresholds: strains below approximately 200 με (disuse range) trigger bone resorption; strains between 200 and 1,500 με (adapted range) maintain bone mass; strains between 1,500 and 3,000 με (mild overload) stimulate bone formation; and strains exceeding 3,000 με (pathologic overload) cause damage or fracture (23). In the context of fracture healing, these concepts suggest that the mechanical environment must evolve in parallel with tissue maturation. Reverse dynamization directly applies these mechanobiological principles by initially allowing controlled micromotion to create a mechanical environment favorable for callus formation and chondrogenesis, before increasing fixation stiffness as the callus matures and requires progressively lower levels of deformation to support mineralization and ossification (24).

Importantly, the tolerance of healing tissues to mechanical strain is not static but evolves over time as tissue composition and stiffness change, providing a strong biological rationale for progressively adapting fixation stiffness throughout the healing process. This evolving relationship between biology and biomechanics has been emphasized previously (3) and provides a theoretical foundation for adaptive fixation strategies such as reverse dynamization.

### 2.2 Stability, strain, and fixation strategy

**Figure 1:**
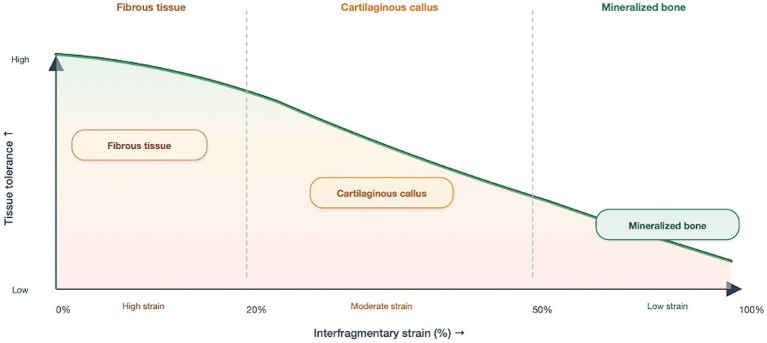
Conceptual representation of the relationship between interfragmentary strain and tissue differentiation during fracture healing. High strain favors fibrous tissue formation, moderate strain supports cartilage formation, and low strain promotes bone formation. This progressive change in tissue strain tolerance provides the biological rationale for reverse dynamization.

**Figure 2:**
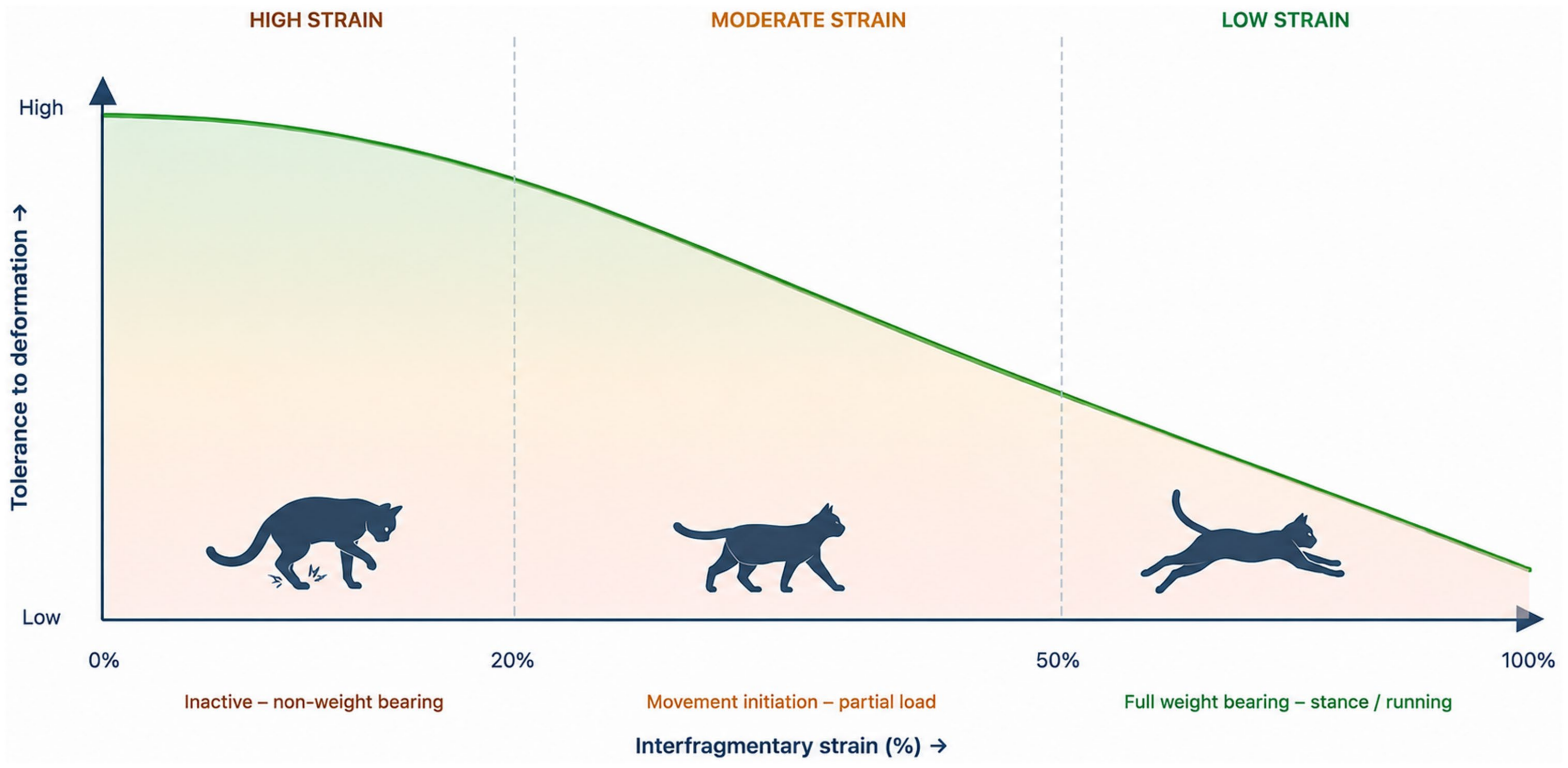
Conceptual representation of the relationship between tissue maturation, interfragmentary strain tolerance, and functional limb use during fracture healing in companion animals. As healing progresses, tissues become less tolerant to deformation while limb loading typically increases from limited weight-bearing to full functional use. This evolving mechanical environment provides a biological rationale for staged increases in fixation stiffness during reverse dynamization. Source: molumen, “cat icons”, Openclipart, uploaded October 6, 2006. Licensed under CC0 1.0 Universal. Modified with Open AI (Chat GPT).

The mechanical requirements of fracture healing therefore vary throughout the repair process. Early fibrous and cartilaginous tissues can tolerate relatively high strain, whereas mineralizing and remodeling bone requires progressively lower interfragmentary motion. This evolving mechanical tolerance highlights a fundamental limitation of fixation strategies that impose constant stability throughout healing. This progression should be viewed as a continuum rather than a binary mechanical state, reflecting the gradual change in tissue composition and tolerance to strain during fracture repair. These evolving mechanobiological requirements and their implications for different fixation strategies are schematically summarized in Figure 1. The progressive relationship between tissue maturation, functional loading, and strain tolerance during fracture healing is further illustrated in Figure 2.

Despite this, fracture fixation strategies have traditionally relied on constructs with relatively constant mechanical properties throughout healing. While rigid fixation effectively maintains alignment, excessive stiffness may suppress the mechanical stimuli necessary for early callus formation during secondary bone healing (3, 4). Experimental and clinical studies have demonstrated that controlled micromotion can enhance callus volume, improve tissue organization, and accelerate the recovery of mechanical strength, provided that instability remains within biologically acceptable limits (7, 23).

### 2.3 Implications for dynamization and reverse dynamization

Conventional dynamization strategies attempt to reconcile these principles by reducing construct stiffness during healing. However, altering stability after several weeks of rigid fixation may occur too late to fully exploit the mechanosensitivity of early healing tissues, and poorly timed destabilization may compromise vascularization or disrupt developing callus (8, 10). These limitations have contributed to the inconsistent clinical performance of traditional dynamization protocols.

Reverse dynamization directly addresses this mismatch between biological progression and mechanical environment. By allowing controlled micromotion during the early stages of healing and subsequently increasing fixation stiffness as mineralization progresses, reverse dynamization aligns construct behavior with the evolving mechanical tolerance of healing tissues (12–14). In controlled experimental settings, reverse dynamization has been associated with earlier callus formation, improved vascularization, and more advanced remodeling compared with static or continuously dynamic fixation strategies (15–17). However, these findings should be interpreted with caution, as the current evidence base derives predominantly from controlled experimental models that may not fully reflect the complexity of naturally occurring clinical fractures. Whether these promising results translate to veterinary patients presenting with variable fracture configurations, soft tissue injuries, and concurrent systemic disease remains unknown.

Together, these mechanobiological principles provide a strong theoretical foundation for reverse dynamization and justify further exploration of this concept in veterinary orthopedic surgery, where fracture healing predominantly relies on secondary bone repair and postoperative loading conditions are difficult to control.

## 3 Clinical perspectives on reverse dynamization in companion animals

Fracture management in companion animals presents specific clinical challenges that complicate precise control of the mechanical environment during healing. In dogs and cats, fracture repair most commonly relies on secondary bone healing, and postoperative loading conditions are difficult to standardize due to species-specific biomechanics, patient behavior, and variable owner compliance (24). Several important differences exist between human and veterinary orthopedic patients that may influence both the feasibility and potential clinical relevance of reverse dynamization. These distinctions include differences in limb loading, postoperative compliance, healing patterns, monitoring strategies, and body weight range. A comparison of key human and veterinary considerations relevant to reverse dynamization is provided in Table 1. Unlike human patients, quadruped animals are capable of functional ambulation despite partial unloading of an affected limb, whether thoracic or pelvic, and can generally avoid weight-bearing on the operated limb during the period of fibrous callus formation with limited functional compromise. This contrasts with human patients, in whom lower limb fractures are considerably more disabling, although the use of crutches may partially compensate for weight-bearing restrictions. Consequently, loading patterns in quadrupeds during the early postoperative period may remain unpredictable and asymmetrical.

**Table 1:** Comparison of human and veterinary considerations relevant to reverse dynamization.

Study (species, model)	Flexible stiffness (N/mm)	Rigid stiffness (N/mm)	Interfragmentary motion (mm)	Transition time
Glatt et al. 2012 (rat femur, segmental defect)	~25	~120	0.5–1.0 → <0.2	21 days
Glatt et al. 2016 (rat femur, +BMP-2)	~25	~120	0.5–1.0 → <0.2	21 days
Glatt et al. 2021 (sheep tibia, osteotomy)	~40	>150	~0.8 → <0.2	28 days
Bafor et al. 2023 (goat, distraction osteogenesis)	Not reported	Not reported	~0.8 → ~0.2	4 weeks

Rigid internal fixation remains the cornerstone of fracture management in veterinary orthopedics and is often selected to protect fracture alignment and provide immediate stability. However, in a clinical context where strict confinement cannot always be ensured, rigid constructs may be exposed to premature or excessive loading once pain subsides. Such loading may exceed the fatigue limits of implants or generate unfavorable interfragmentary strain environments, potentially contributing to fixation failure, delayed union, or nonunion (24). These limitations underscore the difficulty of maintaining a mechanically favorable environment throughout the entire healing period using constructs with constant stiffness.

Interestingly, concepts emphasizing controlled flexibility are not entirely new to veterinary orthopedics. Elastic plate osteosynthesis (EPO) promotes biological fracture fixation through the use of relatively compliant constructs designed to preserve controlled interfragmentary motion while maintaining alignment and overall stability (25, 26). Clinical reports in growing dogs demonstrated rapid callus formation and favorable healing outcomes when long, flexible Veterinary Cuttable Plates were applied using bridge plating principles with limited screw density. Although EPO does not incorporate the staged increase in stiffness that characterizes reverse dynamization, both approaches share the fundamental concept that fracture healing may benefit from a balance between mechanical stability and biological stimulation rather than maximal construct rigidity alone. In this regard, reverse dynamization may be viewed as an extension of existing biologic fixation philosophies, incorporating temporal modulation of construct stiffness in an attempt to better match the evolving mechanical requirements of healing tissues.

Within this context, reverse dynamization may offer a conceptual framework that aligns mechanical stabilization more closely with both biological healing requirements and real-world clinical conditions. By allowing a more permissive mechanical environment during the early postoperative phase, reverse dynamization may accommodate controlled micromotion while animals naturally modulate limb use in response to discomfort. As healing progresses and limb loading increases, a staged increase in construct stiffness could help protect the mineralizing callus during later phases of repair (Figure 2). Importantly, this approach does not assume unrestricted early weight-bearing but rather leverages the intrinsic capacity of companion animals to adjust limb use during recovery.

From a clinical perspective, reverse dynamization may be particularly relevant in fracture scenarios where the mismatch between biological healing requirements and static fixation strategies is most pronounced. Such situations may include comminuted diaphyseal fractures managed with bridging constructs, metaphyseal fractures in growing animals, distraction osteogenesis procedures, fractures in large or highly active dogs, or cases in which postoperative loading conditions are difficult to control. These examples are intended to illustrate clinical situations in which the mechanical environment evolves substantially during healing and should be viewed as hypothesis-generating rather than treatment recommendations.

To date, no clinical studies have evaluated reverse dynamization in veterinary patients. Evidence for its potential relevance is derived primarily from preclinical studies in small and large animal models, which have demonstrated accelerated callus formation, improved tissue organization, and enhanced mechanical strength when fixation stiffness is modulated over time (12, 15–17). While these findings are encouraging, their translation to clinical veterinary fracture management must be approached with caution, as experimental models typically involve controlled osteotomies and standardized loading conditions that differ substantially from clinical fracture scenarios.

It is also important to recognize that the absence of published negative or neutral results should not be interpreted as evidence of universal efficacy. The possibility of publication bias cannot be excluded, as studies reporting neutral or unfavorable outcomes may be less likely to reach publication. Consequently, the current evidence base should be interpreted with appropriate caution. Future veterinary studies should incorporate clearly defined success and failure criteria and should aim to report outcomes comprehensively, irrespective of whether results support or challenge the proposed benefits of reverse dynamization.

From a practical standpoint, the implementation of reverse dynamization in companion animals would require fixation systems capable of predictable and adjustable modulation of construct stiffness. External fixation systems, particularly circular or modular constructs, provide a feasible platform for staged mechanical adjustment and are already used in selected veterinary cases (7, 27). In addition, advances in internal fixation technologies, such as dynamic locking screws and micromotion-based plate constructs, suggest potential avenues for achieving controlled early flexibility followed by increased stability without the need for additional surgical intervention (23, 28). However, defining optimal stiffness ranges, timing of transitions, and postoperative management protocols remains a significant challenge.

Overall, reverse dynamization should currently be regarded as an evolving concept rather than a defined clinical strategy in veterinary orthopedics. Further prospective clinical studies will be required to determine its safety, feasibility, and reproducibility, as well as to identify fracture types and patient populations in which adaptive modulation of fixation stiffness may offer meaningful clinical benefit. In addition to radiographic consolidation, future investigations should evaluate functional recovery, tolerance to loading, and fixation-related complications, as these outcomes ultimately determine clinical success in veterinary fracture management.

## 4 Adaptive fixation devices and technological considerations

The clinical implementation of reverse dynamization relies on fixation systems capable of predictable and controllable modulation of mechanical stability over time. Importantly, reverse dynamization should be viewed as a biomechanical principle rather than a fixation method. Although current technologies provide only limited opportunities for deliberate modulation of construct stiffness during healing, future clinical implementation may rely on a variety of adaptive fixation strategies capable of aligning construct behavior with the evolving biological and mechanical requirements of fracture repair. Unlike static constructs, adaptive fixation devices are designed to interact dynamically with the evolving mechanical environment of the healing bone, offering a potential means of aligning construct behavior with the temporal progression of tissue differentiation.

Although reverse dynamization is often discussed conceptually, several experimental studies have reported quantitative mechanical parameters associated with successful implementation of the technique. Key values for construct stiffness, interfragmentary motion, and timing of stiffness transitions are summarized in Table 2. These values should be interpreted as conceptual reference points rather than prescriptive clinical targets for veterinary patients, given substantial differences in body size, bone geometry, fixation constructs, and loading conditions between experimental models and companion animals.

**Table 2:** Summary of quantitative mechanical parameters reported in preclinical reverse dynamization studies.

Consideration	Human orthopedics	Veterinary orthopedics (dog/cat)	Implication for reverse dynamization
Limb loading	Bipedal: weight-bearing impossible without crutches or total offloading	Quadrupedal: partial unloading possible without assistance; patient can use the other three limbs	Early flexible phase is better tolerated, but risk of sudden excessive loading is higher
Postoperative compliance	Moderate to high (patient understanding and cooperation)	Highly variable (entirely owner-dependent; confinement often difficult to enforce)	Progressive stiffness modulation may help accommodate variable postoperative loading conditions
Bone healing pattern	Primary (direct) healing common in many settings	Secondary (callus) healing predominant in routine veterinary practice	Reverse dynamization may be conceptually compatible with this predominant healing pathway
Monitoring of consolidation	Serial radiographs, CT, ultrasound elastography, implantable sensors	Radiographs under sedation/anesthesia; advanced imaging not routinely available in all practice settings	Need for simple, robust transition criteria based on fixed time points rather than complex parameters
Body weight range	Narrow (typical adult: 50–100 kg)	Very wide (2 kg to over 80 kg depending on breed)	Mechanical parameters may require adaptation across a broad range of patient sizes

External fixation systems currently represent one of the most adaptable platforms for staged modulation of construct stiffness. Circular and modular external fixators allow gradual adjustment of frame configuration, axial stiffness, and load sharing without additional surgical intervention. Experimental and computational studies have demonstrated that frame rigidity can be systematically altered through modifications in ring configuration, strut geometry, or pin number and distribution, thereby influencing interfragmentary motion and strain patterns (7, 27, 29). These characteristics make external fixators a practical tool for exploring reverse dynamization principles in veterinary patients, particularly in situations where postoperative mechanical conditions are expected to evolve substantially.

Advances in internal fixation technologies have also introduced new possibilities for controlled micromotion. Dynamic locking screws (DLS) permit near-cortex motion while maintaining overall construct stability and have been shown to promote increased periosteal callus formation and improved biomechanical properties in experimental models (28). Similarly, plate constructs incorporating elastic or active elements have been developed to allow symmetric axial motion while limiting shear and torsional instability. In controlled animal studies, such active plate systems have demonstrated faster and more robust fracture healing compared with conventional rigid locking plates (30).

More recently, micromotion-based fixation systems and hybrid constructs have been proposed as a means of fine-tuning interfragmentary strain within biologically favorable ranges. Reviews of emerging fixation technologies highlight growing interest in devices that balance stability with controlled flexibility, either through material properties, geometric design, or adjustable interfaces (31, 32). While these approaches are conceptually aligned with reverse dynamization, most currently available systems provide either constant or passively dynamic behavior rather than deliberate, stage-dependent modulation of stiffness.

Despite these technological advances, several challenges remain before adaptive fixation can be routinely applied in veterinary orthopedics. Defining optimal stiffness thresholds, timing of mechanical transitions, and acceptable ranges of micromotion for different fracture configurations and anatomical locations remains an area of active investigation. In addition, veterinary patients encompass a wide range of body sizes, activity levels, and loading behaviors, complicating the standardization of device design and mechanical calibration. Cost, surgical complexity, and learning curves associated with advanced fixation systems may further limit widespread adoption.

At present, adaptive fixation devices should therefore be viewed as enabling technologies rather than definitive solutions. Their primary value lies in providing platforms through which reverse dynamization principles can be investigated and refined in clinically relevant settings. Future developments incorporating sensor technology, real-time load monitoring, or adjustable fixation systems may further enhance the feasibility of individualized fracture care in veterinary patients.

## 5 Challenges and future perspectives

Although the preclinical studies cited in this review report favorable outcomes with reverse dynamization, several methodological limitations must be acknowledged. Most studies employed controlled osteotomy or critical-size defect models in healthy laboratory animals, conditions that differ substantially from the complexity of naturally occurring clinical fractures. Sample sizes were generally limited, independent replication remains sparse, and some influential studies combined reverse dynamization with osteoinductive adjuncts such as recombinant human BMP-2, making it difficult to isolate the specific contribution of mechanical modulation. Furthermore, the literature contains very few neutral or negative reports, raising the possibility of publication bias. Importantly, clinical evaluation of reverse dynamization in veterinary patients remains absent from the current evidence base, limiting direct extrapolation of experimental findings to routine clinical practice.

Despite increasing experimental interest in reverse dynamization, several challenges must be addressed before this concept can be translated into routine veterinary orthopedic practice. A primary limitation lies in the heterogeneity of clinical fracture scenarios encountered in companion animals. Unlike standardized osteotomy models commonly used in experimental studies, clinical fractures often involve variable configurations, degrees of comminution, soft tissue injury, contamination, or concurrent disease, all of which can substantially influence both biological healing potential and mechanical stability (4, 24). These factors complicate direct extrapolation from preclinical models to clinical patients.

Another major challenge concerns the definition of appropriate mechanical thresholds. Reverse dynamization relies on the assumption that construct stiffness can be modulated in a manner that maintains interfragmentary strain within biologically favorable ranges as healing progresses. However, optimal strain magnitudes and the timing of stiffness transitions remain incompletely defined and are likely to vary depending on fracture location, fixation method, and patient-specific factors (12–14). While experimental studies provide valuable proof-of-concept data, they do not yet offer clear guidance for clinical decision-making (13).

An additional consideration relates to inter-individual variability in biological response to fracture fixation. Even under comparable mechanical conditions, factors such as age, body mass, activity level, local vascularity, and systemic health may influence healing kinetics and tissue tolerance to mechanical strain (19, 20). This variability further complicates the application of static fixation strategies and supports interest in adaptive approaches capable of responding to evolving biological and mechanical conditions.

In addition to these challenges, reverse dynamization inherently carries potential risks if the mechanical environment is not appropriately matched to the biological stage of healing. Excessive or prolonged micromotion during early repair may compromise endochondral progression and contribute to delayed union or nonunion, whereas insufficient control of deformation, particularly in shear or torsion, could predispose to malalignment or malunion. Conversely, premature increases in construct stiffness may suppress beneficial mechanical stimuli and limit callus maturation.

Furthermore, some mechanical or biological complications associated with adaptive fixation strategies may be underreported in experimental studies, particularly when radiographic union is achieved despite suboptimal tissue organization or reduced mechanical competence. These considerations underscore the importance of carefully defining mechanical targets, transition timing, and monitoring strategies when translating reverse dynamization concepts into clinical practice.

Practical considerations also limit widespread implementation. Many fixation systems capable of staged stiffness modulation require advanced surgical expertise, careful postoperative management, and increased financial investment. These constraints may restrict their use to selected referral settings rather than general practice (7, 27, 29). In addition, veterinary patients span a wide range of body sizes and activity levels, further complicating device scaling and mechanical calibration (31).

From a research perspective, the current evidence base remains heavily weighted toward preclinical models. Studies in rodents, sheep, and goats have demonstrated accelerated healing, improved callus quality, and enhanced mechanical strength under reverse dynamization protocols (15, 17). However, these models typically involve controlled osteotomies and standardized loading conditions that differ substantially from real-world clinical environments encountered in veterinary practice. Prospective clinical studies in client-owned veterinary patients will therefore be necessary to assess safety, feasibility, and reproducibility, as well as to identify fracture types and patient populations most likely to benefit from this approach. Importantly, future studies reporting neutral or negative outcomes will be equally valuable in refining mechanobiological models and in defining the limitations of reverse dynamization in veterinary orthopedic practice.

Future developments in fixation technology may help address some of these challenges. The integration of sensor-based systems capable of monitoring load transmission, interfragmentary motion, or callus stiffness in real time could enable more individualized modulation of construct behavior during healing (32, 33). Such approaches may facilitate the development of adaptive fixation strategies that respond to biological progression rather than relying on predefined timelines. However, the clinical practicality, robustness, and cost-effectiveness of these technologies remain to be established.

Overall, while reverse dynamization offers a compelling mechanobiological framework, its successful translation into veterinary orthopedics will depend on careful integration of biological principles, biomechanical design, and clinical feasibility. Continued collaboration between clinicians, engineers, and researchers will be essential to refine this concept and to define its role alongside established fracture management strategies (13, 14).

## 6 Conclusion

Reverse dynamization represents an evolving approach to fracture stabilization that seeks to better align mechanical fixation strategies with the biological progression of bone healing. By permitting controlled micromotion during early phases of repair and progressively increasing construct stiffness as mineralization and structural consolidation advance, this concept reflects established mechanobiological principles and challenges static notions of fracture fixation.

Experimental and preclinical studies have provided encouraging evidence that staged modulation of construct stiffness can influence callus formation, tissue organization, and mechanical competence. However, the current body of evidence is derived predominantly from controlled animal models and experimental settings, which differ substantially from the complex and heterogeneous fracture scenarios encountered in veterinary clinical practice. As such, direct translation of these findings to companion animals must be approached with caution.

Importantly, the current evidence base remains limited by the predominance of experimental models, relatively small sample sizes, and the absence of veterinary clinical studies evaluating reverse dynamization under routine practice conditions. Consequently, reverse dynamization should currently be regarded as a promising mechanobiological concept rather than an established clinical strategy in veterinary orthopedics.

This review does not seek to define specific clinical indications or treatment protocols. Rather, it provides a mechanobiological framework through which reverse dynamization can be critically evaluated in the context of companion animal fracture management. The unique biomechanical and practical realities of veterinary fracture care, including quadrupedal locomotion, variable postoperative loading, and predominant reliance on secondary bone healing, provide a compelling rationale for further investigation of staged stiffness modulation. Future veterinary-specific studies will be required to define optimal mechanical thresholds, transition timing, safety, clinical applicability, and potential failure modes.
